# Empyema of Maxillary Sinuses Complicated by Empyema of Contiguous Sinuses

**Published:** 1900-03-15

**Authors:** H. J. Custer

**Affiliations:** Columbus, O.


### EMPYEMA OF MAXILLARY SINUSES COMPLICATED BY EMPYEMA OF CONTIGUOUS SINUSES.

BY H. J. CUSTER, COLUMBUS, O.

Read before the Ohio State Dental Society, Dec. 6, 1899.

On account of the frequency with which the various lesions of the maxillary sinus are supposed to result from diseased teeth, the dentist is often the first one to examine the case. For this reason it is well that the dentist be able to diagnose and prognose the case, even though he may not choose to treat it.

In a paper presented to this society on a former occasion the writer discussed the treatment of maxillary empyema when complicated by various conditions within the antrum; this paper will, therefore, be devoted to complications exterior to the antrum, or, still more specifically, to the differential diagnosis of empyema of the frontal, ethmoidal, sphenoidal and maxillary sinuses, with special regard to the latter condition when other sinuses are involved.

From recent anatomical investigations we find that the maxillary sinus is a cavity of irregular size, shape and position. It usually stands over at least some portion of the alveolar process, but may be small and entirely external to the alveolar arch, so that the roots of some posterior teeth may enter the nasal fossa.

A portion of the antral floor is usually below that of the nasal passage; the entire floor of the antrum may rarely be higher than the nasal floor, so that, as Cryer has demonstrated, an opening through the canine fossa would enter the nasal passage, where the antrum is small and situated well above and external to the alveolar arch.

This irregularity should also be remembered in transillumination. The greatest area of thin bone is not in the canine fossa, but forms the internal wall of the antrum, so that in alveolar abscess the contour of the canine fossa is modified, while in confined suppuration of the antrum the internal wall will often bulge before the canine fossa is disturbed.

The natural opening of the antrum is usually as far back as the first molar and opens between the middle meatus and usually the upper part of the antrum. But its position and size is not constant. There may be several openings, or even none at all.

Between the lower halves of the orbital cavities lie the ethmoid cells, just to the rear of and below which is the sphenoidal sinus. In rare instances the crooked canal draining the frontal sinus opens direct into the maxillary sinus; but it usually opens into the middle meatus a little anterior to and above the ostium maxillaire, and in the same regions are the openings of the anterior ethmoid cells. The openings of the posterior ethmoid cells and sphenoidal sinus are in the superior meatus.

We have in the maxillary, sphenoidal, frontal and ethmoidal sinuses or cells a set of cavities the lining membrane of which is practically identical, while their various diseases, along with their causes, are also much the same.

When we further consider that the outlets of these several sinuses occur within one small area that is subject to great variation, it is not surprising that two or more sinuses may be involved at the same time; or, that when only one cavity is involved we may at times be perplexed to locate, so far as the cause of empyema of any of the sinuses is a factor of the diagnosis, acute and chronic rhinitis, eruptive fevers, surgical and accidental traumatism, insects, foreign bodies, and erysipelas may cause empyema in any of the sinuses without much choice of selection, with the exception that the sphenoidal sinus is less exposed to traumatism and erysipelas and that the maxillary sinus has additional exposure of hydrargysm and dental lesions.

Acute empyema of the maxillary sinus is characterized by a variable amount of pus coming from the middle meatus and sometimes escaping into the pharynx, while sleeping and at other times, through the nostril. The discharge is usually light yellow, and the odor may be slight or pronounced. It may be rather continuous or more or less periodical, depending on temporary occlusion of the ostium and certain positions of the head. Dull, heavy, deep-seated pain within the cheek is common and may be frontal or supraorbital pain or a general headache, and, not infrequently, fever, sweating and malais; the cheek is often feverish, tender, and fuller than usual. The conjunctiva is likely to be affected and the lids swollen. The mucous membrane of the canine fossa and posteriorly is deeper in color and the region is tender on percussion. Percussion of the teeth below the antrum usually reveals at least slight periodontitis, and pressure upon the hard palate often finds a tender spot. The mucous membrane of the nasal wall will be congested and tender, especially about the antrum.

In chronic empyema there is less discharge than in the acute form, and in consistency it is probably less thick, the color and odor being about the same. The pain is from none at all to a dull, heavy, uneasy sensation deep within the cheek, and there may be moderate frontal or supraorbital pain or a general headache. Bryant mentions two cases in which the only symptom was supraorbital neuralgia, one case being cured and the other temporarily relieved by opening the antrum. Le Fevre reports one case whose only symptom was occipital headache which ceased on opening the antrum.

When the wall of the canine fossa is thin, there is likely to be tenderness on percussion. Sometimes the teeth below the antrum are perfectly comfortable on percussion or hard pressure, and the hard palate may be just as irresponsive, even though the antrum lies above.

The mucous membrane of the nasal wall is often thick and congested. When pus of either acute or chronic empyema fails to make its escape, the symptoms of the acute form become pronounced. There is intense boring pain in the jaw, often attended by severe pain in almost any part of the head or neck. The cheek is flushed, hot, tender and swollen, and there may be exophthalmos along with visual disturbance.

There may be bulging with crepitation of the canine fossa, but the nasal wall is most likely to bulge first, and it is always very tender. Almost always there is periodontitis, with a tender and perhaps bulging hard palate and inability to open the mouth.

Fistula may open either through the cheek, along the side of a tooth, above the alveolar arch, through the hard palate, into the nasal or orbital cavity, or into the pterygoid or spheno-maxillary fossa. The writer has seen fistula established in all those positions excepting the two last. There is fever, colliquative sweating, and the prostration is sometimes alarming to friends of the patient.

*The frontal sinus*—In acute empyema of this cavity there is often a blush over the region which is the seat of intense pressing pain, which may radiate, especially along the supraorbital nerve.

Pressure upon the frontal eminence in the upper inner angle of the orbit, and often along the supraorbital nerve, will cause much pain. There is photophobia, conjunctival injection, periocular edema, and perhaps some exophthalmia. The pain is increased by inclining the head downward or by coughing and sneezing. The discharge is usually a light yellow mucopurulent and without much odor, and first appears rather high and well forward in the middle meatus. The discharge is likely to be more or less periodical, recurring one or more times daily and frequently just after sneezing, when the pain quickly remits. According to Myles there is dullness in percussion. I have percussed many times, but have never been able to detect more than ordinary dullness.

In chronic frontal empyema the symptoms are much the same as in the acute form, only less pronounced. There is not likely to be a discoloration over the sinus, except, perhaps, during cold or damp weather, when an exacerbation is liable to occur. The patient becomes apathetic, depressed, forgetful and incapacitated for business. When the pus is confined the acute symptoms are pronounced and there is bulging in the frontal corner of the orbit and perhaps a fistula opening externally or into the orbital or cranial cavity.

*The ethmoid region*—Acute empyema of the ethmoid cells is so often accomplished by influenza or some form of rhinitis that it is seldom recognized as a separate condition. When present there is a painful tension between the eyes, and this is increased when the head is lowered and by coughing and sneezing. There is febrile disturbance and a rather copious discharge of inodorous pale yellow mucopurulent fluid from the middle meatus where the anterior cells are involved, and from the superior meatus where the posterior cells are involved.

In chronic empyema there is often discoloration around the inner canthus and there is a disagreeable, uneasy sensation between the eyes. The discharge alters in quality and quantity from time to time and occasionally contains shreds of soft tissue or particles of bone. When of some duration, a probe in the ethmoid region will often detect denuded, crumbling bone. When the suppuration is confined there is often a bulging at the inner canthus along with other ocular disturbance and symptoms of confined pus, much the same as those of the other sinuses. A fistula may open externally or into the orbital or cranial cavity.

Empyema of the sphenoidal sinus on account of its location is difficult to recognize. Recent investigation, however, shows the condition to exist more often than was formerly supposed. In the acute form the symptoms are much obscured by attending inflammation; but there is is liable to be a sense of weight and pressure deep in the middle part of the head—a vague sense of occipital distress and dull headache. There may be temporal or deep-seated orbital pain, increased by sudden movement of the head, and pain in a part or all of the trigeminal nerve. Sometimes there is tinnitis aurium and vertigo; there is more or less mucopurulent discharge of varying odor and consistency and escaping far back in the superior meatus. This discharge usually passes into the pharynx instead of through the nostril. When the condition is chronic the symptoms are much the same as in the acute form, only milder; but the discharge may contain particles of crumbling bone. Kyle is certainly correct in picturing the mental condition as one of lassitude and depression, with heavy eyes, sleep disturbed, breath sour, stomach disordered, and the general appearance that of suppurative cachexia. When pus is confined there is the most intense pain and likely to be marked occular and visual disturbances. Irregular fever and sweats develop, prostration is profound, mental symptoms are marked and cerebral complications may follow.

My own surgical experience with sphenoidal empyema has been limited to one case, which is important as showing the mental phenomena liable to occur when pus lies within close proximity to the brain, the mental condition always being more marked in empyema of upper sinuses than in empyema of maxillary sinus. The patient, a married lady forty-six years of age, residing near this city, had for fifteen years complained of nasal trouble, and for the past four years there was every three or four months a sudden free discharge of moderately fetid, pale mucopurulent fluid into the nose and pharynx. This discharge was always preceded by three or four weeks of intense, boring pain deeply situated in the median line. In the words of the patient, there was at times "a sensation as of something moving within the head." "The head nearly always feels feverish and painful." There was disturbed sleep, impaired memory, heavy eyes, depression and hebetude, also an inclination to self-destruction, which was specially marked on lying down. Slight conjunctival congestion was the only ocular disturbance that could be attributed to the empyema, though the entire right side of the face was fuller than the opposite, this condition seeming to be from atrophy of the left side rather than disturbance of the right. The general appearance was that of some grave disorder. Prior to February 1, 1899, I had by two operations removed a number of polypi, a middle turbinated bone and a part of the septum, and on the above date I opened and curetted the sphenoidal sinus, removing a small amount of fetid fluid and some spongy membrane. Syringing and tonic treatment was adopted, and general improvement soon followed. Ten months have now passed and the patient has been comparatively free from pain, excepting three months ago, when the former trouble threatened to repeat itself; but the pain, which did not equal the original, subsided without a discharge occurring. The patient has increased in weight and is much improved both mentally and physically. For the present purpose it is not necessary to differentiate between empyema of the frontal, ethmoidal or sphenoidal sinuses, but we do wish to differentiate between empyema of any or all of these sinuses and that of maxillary sinus. From the foregoing symptoms it is evident that there will not be much difficulty in locating a confined suppuration, unless it be that of the sphenoidal sinus. The recognition of the acute form will be more difficult, while the chronic conditions will at times tax the most competent. To this condition special attention will be given.

Even though, from symptoms already stated, we are able to diagnose maxillary empyema, should there be a discharge into the pharynx or through the nostril we should always make a careful examination of nasal passages. The same cause may induce empyema simultaneously in two or more cavities, or the empyema may extend by continuity of tissue or by infection from above downward or from below upward.

Any person who is accustomed to looking into mirrors and small cavities, as is the dentist, will not have much difficulty in passing a small mirror into the pharynx and having a good look at the posterior nares, and inspection of the anterior nares will be even less difficult. When pus is seen in the middle meatus it may be from the frontal sinus, from the anterior or middle ethmoid cells, or from the maxillary sinus, even though the head is upright. Wipe away the pus or wait for more. If it appears well forward, under the middle turbinated bone, it is probably from the infundibulum, especially if a probe can be inserted into a canal. Discharge from the ethmoid cells seems to come from all around above the antral opening and usually has less odor than discharge from the antrum. Pus from the antrum usually has an odor that is perceived by the patient and his friends; pus from the ethmoid cells often is without odor, but even if there was odor the patient would likely be unconscious of it.

In chronic empyema of the ethmoid cells there are often particles of soft tissue and bone in the discharge, and a probe in the ethmoid region will often detect denuded, crumbling bone. For this manipulation the keen tactile sense of the dentist probably has no superior. A blunt probe of proper curvature may sometimes be inserted through the ostium maxillæ for about an inch; this will serve to locate the opening while studying the flow of pus. To facilitate matters a 4 percent spray of cocain, followed by a 3 percent spray of suprarenal capsule extract, will so anæsthetize and contract the tissues that inspection and manipulation will be easier, and pus may now appear where it would not otherwise. Before using these solutions I always study the case well in its original conditions. Pus coming from the posterior ethmoidal cells or sphenoidal sinus does not often run into the region of the antral opening unless there be polypi or deformities directing it out of its usual course. It is best seen by posterior retinoscopy and appears in the space between the posterior end of the middle turbinated bone and the septum.

After placing the head in such a position as to make the ostium the dependent part of the antrum, it is well to wipe away the pus and wait for its reappearance. If pus is in the antrum it nearly always escapes, while pus from the upper sinuses will not appear. Should the pus not now escape from the ostium, it is well to irrigate with sterilized water, for the pus may be too carious to escape even though the osteum be open.

In some cases I have used peroxid of hydrogen for diagnostic purposes, but only well diluted and with a free opening to avoid pressure. Transillumination is a valuable aid in diagnosing empyema, especially that of maxillary sinus, and I have had good results in some cases of frontal empyema.

To transilluminate successfully the room should not only be darkened but should be absolutely dark. A four-candle lamp will give ample light. When the walls of a healthy antrum are of average position and thickness the antral region has a dull glow, with a bright, semilunar region just below the eye. There is often a pupillary glow and a subjective sensation of light within the eye.

When there is pus within the antrum the entire region is darker and the semilunar area is specially obscure. The pupillary glow is reduced or absent, as is the subjective sensation of light. My own experience agrees with that of other observers in that the subjective sensation of light is well worth considering.

I am not aware of any other person having called attention to the importance of the position of both the head and the light within the mouth while transilluminating. I always use both head and light in various positions, and have noted two cases in which by placing the light well back within the mouth and tilting the head well forward there was a bright semilunar area, pupillary glow and subjective sensation of light, and yet I took pus from the antrum after examination.

*Treatment*—Confined suppuration in any of the sinuses requires surgical treatment. Acute empyema of the upper sinuses may recover by medical treatment, or even spontaneously, but acute empyema of the maxillary sinus requires irrigation, if not through an artificial opening, at least through the natural opening. Chronic empyema of the antrum, in the opinion of the writer, is best drained through the mouth, and, when not complicated, usually responds well to such treatment. But if there be also chronic empyema of the ethmoid cells or of the frontal sinus, the maxillary drainage will only palliate the case and not cure.

In conclusion, the history of the case, the symptoms, a careful study of the nasal passages, perhaps the use of hydrogendioxide, and transillumination, will usually decide whether the maxillary empyema is void of external complications, in which instance the disease will often yield promptly to treatment by the dentist whose methods of obtaining free drainage are probably superior to that of other practitioners.

DISCUSSION OF DR. CUSTER'S PAPER.

DR. TAFT: My experience has been that in almost every instance where there is a condition of the antrum such as described by the paper, there is always more or less of a marked mental change or impression that is adverse to the physical well-being of the patient, and this is doubtless many a time altogether overlooked.

There is no doubt that this could be utilized in a sense in making a diagnosis. I would like to ask Dr. Custer if he has not found this the case in nearly every instance.

DR. CUSTER: Yes; in my experience I have found it so.

DR. TAFT: This condition is also noticed when the sinuses above the orbit are affected, but it is not so marked as that produced by larger cavities, such as the antrum. The larger surface involved the more general condition of malais.

So I say this should be taken into account. I have no doubt if due discrimination was always made the diagnosis would be very much clearer. Of course we must have the history of every case. Patients can give very much information if correctly questioned, and many times I think diagnosis is faulty because of want of the utilization of facts.

DR. J. R. OWEN: I would like to ask Dr. Custer what stimulating and antiseptic washes he has found most satisfactory in such cases?

DR. CUSTER: I have found boracic acid, also a 2 percent solution of formaldehyde, and sometimes I have used nothing but sterilized water, simply washing out thoroughly, especially if I have good access.

DR. H. A. SMITH: Why is it that more of us are not interested as is our friend Custer? When we anticipate a lesion of this sort we confine our investigations to the maxillary sinus, leaving out of consideration the disorders in the other related sinuses. Dr. Cryer, I think, has shown the importance of this very conclusively. We must examine the other sinuses. We are not broad enough for that as yet. It is a hopeful sign to have such a paper as this read here, and should impress upon us the importance of a conclusion on such subjects or cases. It would be especially profitable to us if in Cincinnati we had such a specialist as Dr. Custer.

The point which Dr. Taft brought out is excellent; we fail to take into account the mental condition of patients too often in our diagnosis. If you go into an insane asylum to-day you will find people insane wholly because they have empyema in the antrum or related sinuses, and those in charge of the patients do not recognize the impressions these diseases make. We often have cases where we suspect empyema of the antrum and treat it without success, and we never investigate for inflammatory conditions in the frontal sinuses. We do not take the mental conditions into consideration at all. The patient may complain of a different location of pain, not in the antrum. When this is the case, it is beyond the scope of our present investigation. The continuity of these sinuses alone should lead us to investigate further. Why is it we do not consult with each other? We must do these things in order to get our education. I congratulate our friend Custer that he had a laboratory education in the dental profession.

Dr. M. H. Fletcher: There is one thing which was not discussed by the essayist which would probably have been discussed if he had chosen to take it in, and that is the etiology of these cases. It is a question in my mind as to whether the diseases of the maxillary sinuses are to blame—that is, whether they are more largely caused by the teeth or by intra-nasal disorders. It has been discussed a great deal by those interested in this topic. Five hundred skulls were examined by me with this idea in view. It was a remarkable fact to me that so few teeth projected into the antrum. In the majority of cases there was only a thin layer of bone, and sometimes much cancellous even above the apices of the teeth. On the other hand, there were many found in which the teeth penetrated into the antrum. In these cases the roots are simply covered with soft tissue during life. Assuming this to be true in the case of empyema of maxillary sinus, with engorgement and finally becoming chronic, the floor of the antrum would naturally give way first because of constant covering of pus; so, if this be true, the nerve and blood supply to the teeth, which hold such a position to the floor of the antrum, would become destroyed in the course of time.

The symptoms as cited in the case here presented—the teeth on the side affected were all sore and involved. Now generally, if I see the maxillary sinus filled with pus, I look further than the teeth for the cause. The pressure on the teeth which had no bony covering would push the teeth down from the socket and would naturally produce inflammation of peridental membrane. That patient appearing in dental office for diagnosis, the operator would examine the teeth and discover considerable inflammation about roots of teeth involved, and he would be very apt to say there is the cause of the trouble. I know that a few years ago it was thought that the teeth were more largely to blame than any other cause for these conditions. Looking at it from this standpoint, the teeth would be blamed immediately where it is largely the reverse. I call this to your attention, that most of these cases are looked upon as strictly dental cases and we examine teeth only. If the tooth is tender on pressure, you would say there must be somethihg wrong with the tooth.

I would first investigate the intra-nasal conditions. Take the history of the case, find out what the trouble is, and be sure you do not have intra-nasal trouble with the maxillary engorgement. I am free to admit that there are many cases of antral trouble coming from the teeth, but my own experience in treating these cases reveals the fact that when they are due to dental abcesses recovery is very rapid, and more so than when caused by intra-nasal troubles.

Method of diagnosis: Transillumination. I had occasion to speak of that yesterday. I have found nothing in my own practice which has given me more satisfaction and a more thorough means of diagnosis than that of transillumination. It is not a hard matter to arrange for this work. A simple lamp socket, holding a three to six candle power lamp, can be operated by one who has the skill to be a good dentist. If not on the market, you can make one yourself. If the lamp burns out, it is a matter of ten or fifteen cents to get another. It is well worth your while to have the apparatus at hand to diagnose these troubles. The prognosis of these cases many times is very bad. The healing or recovery I believe that Dr. Custer will bear me out in saying they are very slow, especially if they are caused by intra-nasal disorders. If they are from the teeth, they recover much more rapidly.

Dr. Custer: Referring to the matter touched upon by Dr. Taft, as to mental symptoms exhibited, it has been my experience in chronic empyema there is more pronounced mental disturbances from the antrum than any other sinus, because of the larger amount of surface and of mucous membrane exposed, consequently of more absorption and because drainage is not so good here as in other sinuses. In confined suppuration I rather think the mental symptoms will be a little more pronounced in the upper sinuses on account of proximity to cranial cavity.

In the prognosis of case as mentioned by Dr. Fletcher it is always well to consider the nasal passages along with the teeth. Maxillary lesions depending on dental troubles respond more readily than those depending on nasal troubles.

QUESTION: How do you determine when the sinus is full of pus?

ANSWER: By transillumination. It gives a different shadow.

